# Identification of GROWTH-REGULATING FACTOR transcription factors in lettuce (*Lactuca sativa*) genome and functional analysis of *LsaGRF5* in leaf size regulation

**DOI:** 10.1186/s12870-021-03261-6

**Published:** 2021-10-23

**Authors:** Bin Zhang, Yanan Tong, Kangsheng Luo, Zhaodong Zhai, Xue Liu, Zhenying Shi, Dechun Zhang, Dayong Li

**Affiliations:** 1 National Engineering Research Center for Vegetables, Beijing Vegetable Research Center, Beijing Academy of Agriculture and Forestry Science, Beijing, 100097 PR China; 2 Beijing Key Laboratory of Vegetable Germplasm Improvement, Beijing, 100097 PR China; 3 Key Laboratory of Biology and Genetic Improvement of Horticultural Crops (North China), Ministry of Agriculture and Rural Affairs of the P. R. China, Beijing, 100097 PR China; 4 Biotechnology Research Center, China Three Gorges University, Yichang, 443002 PR China; 5 College of Life Sciences, Shandong Normal University, Jinan, 250014 PR China; 6 CAS Center for Excellence in Molecular Plant Sciences, Shanghai Institute of Plant Physiology and Ecology, Chinese Academy of Sciences, Shanghai, 200032 PR China

**Keywords:** Lettuce, GROWTH-REGULATING FACTOR, Leaf development, MicroRNA396, Genome-wide analysis

## Abstract

**Background:**

GROWTH-REGULATING FACTORs (GRFs), a type of plant-specific transcription factors, play important roles in regulating plant growth and development. Although *GRF* gene family has been identified in various plant species, a genome-wide analysis of this family in lettuce (*Lactuca sativa* L.) has not been reported yet.

**Results:**

Here we identified 15 *GRF* genes in lettuce and performed comprehensive analysis of them, including chromosomal locations, gene structures, and conserved motifs. Through phylogenic analysis, we divided *LsaGRFs* into six groups. Transactivation assays and subcellular localization of LsaGRF5 showed that this protein is likely to act as a transcriptional factor in the cell nucleus. Furthermore, transgenic lettuce lines overexpressing *LsaGRF5* exhibited larger leaves, while smaller leaves were observed in *LsaMIR396a* overexpression lines, in which *LsaGRF5* was down-regulated.

**Conclusions:**

These results in lettuce provide insight into the molecular mechanism of *GRF* gene family in regulating leaf growth and development and foundational information for genetic improvement of the lettuce variations specialized in leaf character.

**Supplementary Information:**

The online version contains supplementary material available at 10.1186/s12870-021-03261-6.

## Background

As one of the plant-specific transcription factors, GROWTH-REGULATING FACTORs (GRFs) are important regulators in plant growth and development. The first identified GRF functions on gibberellic acid (GA)-induced stem elongation [1]. In various studies, the functions of *GRF* genes were found in development of leaf, stem, seed and root by regulating cell proliferation or cell expansion to form large organs [1–8], other new functions in flowering, stress response and plant longevity were uncovered recently [9–15]. Two conserved domains, the QLQ (Gln, Leu, Gln, InterPro: IPR014978, PFAM: PF08880) domain, which is considered to be a protein-protein interaction domain, and WRC (Trp, Arg, Cys, InterPro: IPR014977, PFAM: PF08879) domain, which is supposed to be involved in DNA binding, are found in the N-terminal of GRF family proteins [1, 2, 8, 16–18].

*GRF* family genes are found to be involved in plant growth, development and regeneration. The seedlings with overexpressed miR396-resistant *AtGRF1* or *AtGRF3* showed shorter roots [9]. *BnGRF2* could increase seed weight and oil content by upregulating the expression of chloroplast-related genes in rapeseed (*Brassica napus*) [19]. In rice (*Oryza sativa*), *OsGRF4* played an important role in grain weight [20, 21]. GRF proteins form functional transcriptional complex with the transcription cofactor GRF-Interacting Factors (GIF) [22], and GRF-GIF chimeras could dramatically boost regeneration in various species [23]. Furthermore, most *Arabidopsis thaliana GRF* genes play important roles in leaf size control [6–8]. Among the nine *AtGRF* genes, six *GRF* genes, *AtGRF1*, *AtGRF2*, *AtGRF3*, *AtGRF4*, *AtGRF5* and *AtGRF9*, were proved to function in leaf development [3, 4, 12]. Overexpression of *AtGRF1* and *AtGRF2* respectively cause larger leaves with increased cell size, while *atgrf1/2/3* triple mutant showed smaller and narrower leaves [2]. Overexpression of *AtGRF5* exhibit bigger leaves due to increased cell number but not cell size [3]. The function of *AtGRF5* could not be replaced by other *AtGRFs*, though some functions of them overlap [3, 12]. *AtGRF9* contribute to determining final leaf size, although it has a minor role in cell proliferation [3, 24].

miRNAs, about 20 nucleotides (nt) in length, are single-strand, non-coding, small-molecular-weight RNAs, which could regulate gene expression through target mRNA cleavage or/and translational inhibition [25–27]. Genome-wide analyses reveal that *GRFs* and a few bZIP transcription factor genes are the major targets of microRNA396 (miR396) [28]. miR396 shares nearly perfect sequence complementarity with the transcript of WRC motif in seven members of the *AtGRF* genes, except for *AtGRF5* and *AtGRF6* in *Arabidopsis thaliana* [29, 30]. Correspondingly, miR396a and miR396b regulate leaf growth and development by repressing the expression of *AtGRFs* [29, 30]. MiR396a regulates flower formation, including sepal-petal identity, by regulating the expression of *GRF* gene [15]. Additionally, the miR396-AtGRF module could also regulate adaxial–abaxial (Ad-Ab) polarity formation during leaf morphogenesis [31].

The ever-developing whole-genome sequencing technology, identifies *GRF* genes in various plant species, such as *Arabidopsis thaliana* [2], *Oryza sativa* [16], *Zea mays* [32], *Brassica rapa* [33], *Solanum lycopersicum* [34], *Pyrus bretschneideri*, *Vitis vinifera* [35], *Brassica napus* [36], *Nicotiana tabacum* [37] and *Populus trichocarpa* [38]. Lettuce (*Lactuca sativa* L.) is an important leafy vegetable, the leaf size of which has significant meaning for production. The whole genome sequence of lettuce cultivar ‘Salinas’ has been recently released [39]. However, the *GRF* gene family in lettuce has not been evaluated yet. In this study, we identified 15 *GRF* genes in lettuce and respectively named them based on their chromosomal location. They were divided into six groups according to phylogenic tree of LsaGRFs and AtGRFs. To investigate the transcriptional factor character, LsaGRF5 was specifically studied for its subcellular location and transcriptional activity. In addition, *LsaGRF5* was found to be cleaved as a target of miR396. Using transgenic plants, *LsaGRF5* could enhance the leaf growth in lettuce and *Arabidopsis thaliana* when overexpressed, while smaller leaves were obtained through overexpression of Lsa-miR396 in lettuce. These results may provide a foundation for further elucidation of the function of LsaGRFs and Lsa-miR396 in leaf growth regulation in lettuce.

## Results

### Identification of the *GRF* genes and miR396s in lettuce

**Fig. 1:**
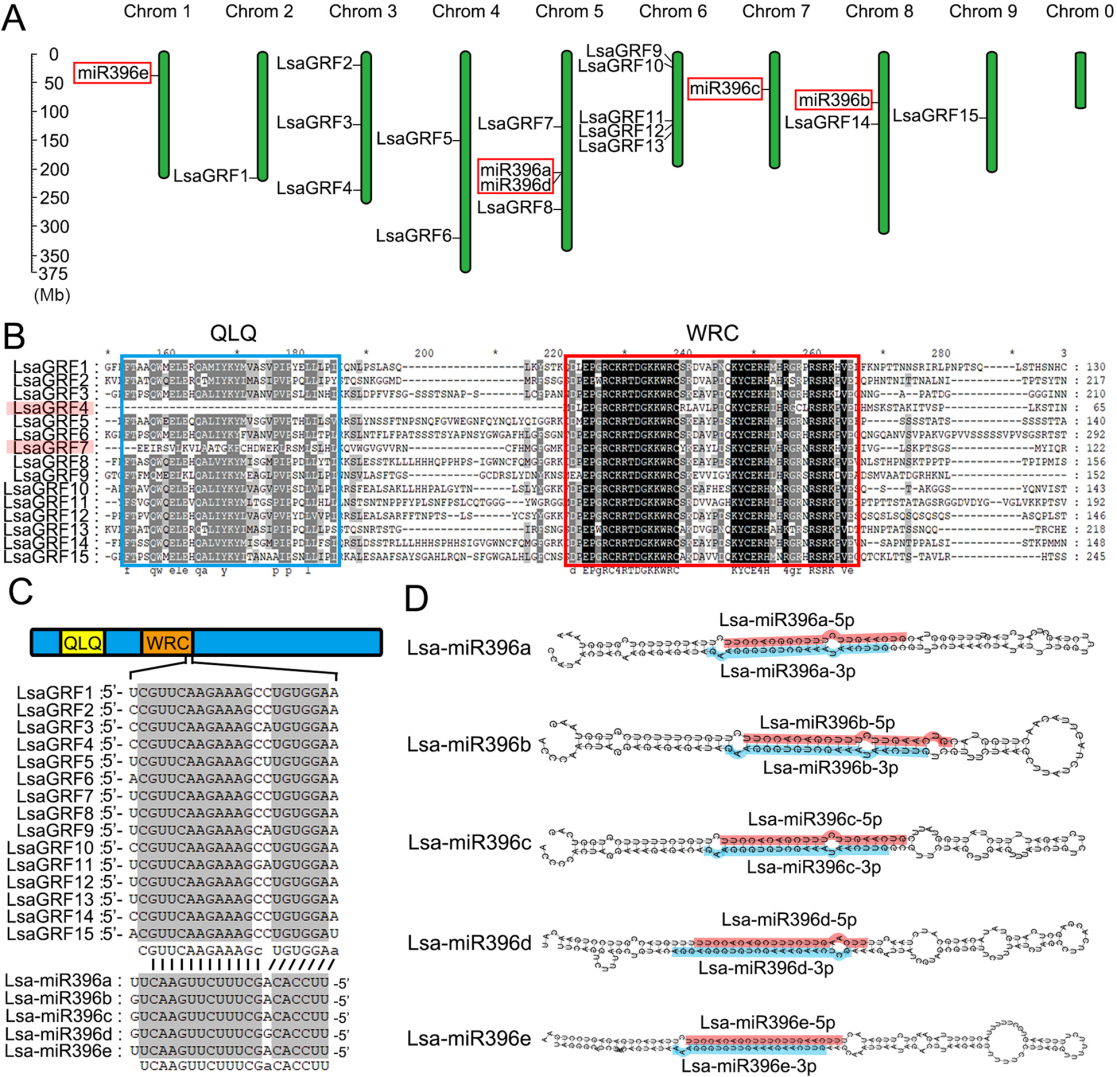
Chromosomal locations and sequence alignments of *LsaGRFs* and Lsa-miR396s. **A** The chromosomal locations of *LsaGRFs* and *Lsa-MIR396s*. *Lsa-MIR396s* were in red rectangles. Chromosome 0 was the sequence which could not be assembled onto any other chromosomes. **B** The amino acids sequences alignment of the conserved domains, QLQ and WRC, of different GRF proteins in lettuce. LsaGRF4 lacking QLQ domain and LsaGRF7 with an incomplete QLQ domain were highlighted red. **C** Scheme representing the *LsaGRF* genes and Lsa-miR396s. The interaction of the miRNA-regulated *LsaGRFs* with miR396 is shown. **D** The stem-loop secondary structures of *Lsa-MIR396s*. The mature 5p-miRNA/3p-miRNA nucleotides sequences were shaded in red and blue, respectively

To identify the *GRF* gene family in lettuce, the *GRF* genes from *Arabidopsis thaliana*, rice and tomato were firstly used as the query sequences for BLASTN searching in lettuce genome database. Thus, thirteen candidate GRF genes were obtained in lettuce. GRF protein contains two conserved functional domains, QLQ and WRC. Therefore, QLQ and WRC were used for the Hidden Markov Model (HMM) search, and another two candidate *LsaGRF* genes were identified. Finally, the amino acids sequences of all fifteen candidate *LsaGRF* genes were applied for BLASTP searching, and no further hits were got. Thus, there are totally 15 *LsaGRF* genes identified in lettuce genome. Based on their respective location on the chromosomes, we designated them as *LsaGRF1* to *LsaGRF15* respectively (Fig. 1A). *LsaGRFs* were distributed on each chromosome of lettuce, except for chromosome 1 and 7, and the most (five *LsaGRFs*) occurred on chromosome 6 (Fig. 1A). The basic information of *LsaGRF1–15*, including gene ID, chromosomal location, length of gene and protein, PI value and exon numbers, was listed in Table 1. Most *LsaGRFs* had 3 or 4 exons, while *LsaGRF4* and *LsaGRF9* had 2 exons and *LsaGRF7* had 6 exons (Table 1). In addition, the predicted isoelectric point (pI) values of LsaGRF preoteins were between 6 and 9, except that the pI values of LsaGRF4 and LsaGRF7 were higher than LsaGRF9 (Table 1). The amino acid sequence alignment of 15 LasGRF was showed in Fig. S1. All of the 15 LsaGRFs contained the conserved WRC domain, but only 13 LsaGRFs had complete QLQ domain. There is no QLQ domain in LsaGRF4, and LsaGRF7 has only an incomplete QLQ domain (Fig. 1B).

**Table 1:** Characteristics of the GRFs in lettuce

Name	Accession No.	Chr	CDS (bp)	Exon No.	Length (aa)	MW (KDa)	pI
LsaGRF1	Lsat_1_v5_gn_2_135541.1	Chr02	990	3	329	36.06	8.76
LsaGRF2	Lsat_1_v5_gn_3_14660.1	Chr03	1227	4	408	44.51	6.24
LsaGRF3	Lsat_1_v5_gn_3_85521.1	Chr03	999	4	332	37.74	8.73
LsaGRF4	Lsat_1_v5_gn_3_133500.1	Chr03	702	2	233	27.17	9.63
LsaGRF5	Lsat_1_v5_gn_4_92941.1	Chr04	951	3	316	36.58	8.81
LsaGRF6	Lsat_1_v5_gn_4_159961.1	Chr04	1551	4	516	56.20	7.70
LsaGRF7	Lsat_1_v5_gn_5_54781.3	Chr05	603	6	200	22.70	9.27
LsaGRF8	Lsat_1_v5_gn_5_141200.1	Chr05	990	3	329	37.54	8.24
LsaGRF9	Lsat_1_v5_gn_6_10680.1	Chr06	459	2	152	16.89	8.66
LsaGRF10	Lsat_1_v5_gn_6_17460.1	Chr06	897	3	298	33.12	6.44
LsaGRF11	Lsat_1_v5_gn_6_70601.1	Chr06	1113	4	370	40.08	8.23
LsaGRF12	Lsat_1_v5_gn_6_75441.1	Chr06	1014	3	337	37.60	8.74
LsaGRF13	Lsat_1_v5_gn_6_81681.1	Chr06	1029	4	342	36.78	7.09
LsaGRF14	Lsat_1_v5_gn_8_86361.1	Chr08	1071	3	356	40.63	8.97
LsaGRF15	Lsat_1_v5_gn_9_78540.1	Chr09	1233	4	410	44.10	7.67

The *GRF* genes are known as the target of miR396s. To date, two *MIR396s* in *Arabidopsis* (*Ath-MIR396s*) and seven *MIR396s* in rice (*Osa-MIR396s*) are identified. There are five *MIR396*s, *MIR396a* to *MIR396e*, in lettuce genome [39]. The phylogenic trees of five *Lsa-MIR396s* and *MIR396s* in lettuce, Arabidopsis and rice were shown in Fig. S2A and B, respectively, based on their stem-loop sequences. The target sequence of miR396s locates at the end of the WRC domain (Fig. 1C). Although the stem-loop structures were totally distinct, five Lsa-miR396s were highly conserved in the mature region with only two nucleotides difference (Fig. 1C and D). Five *Lsa-MIR396*s were located on chromosome 1, 5, 7 and 8 respectively, among which chromosome 5 contained two *Lsa-MIR396s*, *Lsa-MIR396a* and *Lsa-MIR396d* (Fig. 1A). The identified *GRF* genes and miR396s in major species were listed and compared with those in lettuce (Table S1). The number of *LsaGRF* genes was the second largest, and the number of miR396s was comparable to that in rice and tomato.

### Phylogenetic analysis, gene structures and motif divergence of *LsaGRFs*

**Fig. 2:**
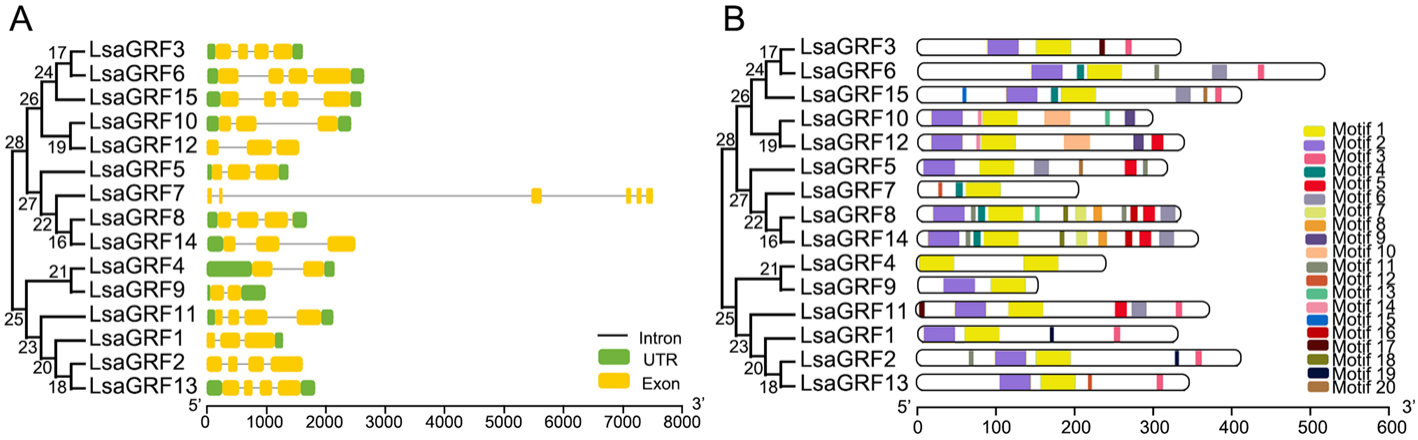
Gene structures and motif composition of LsaGRFs. **A** The gene structures of *LsaGRFs*. **B** The motif composition of LsaGRF proteins with MEME. Twenty motifs are represented by different colored boxes. The same color rectangle in different proteins referred to the same motif. The sizes of motifs are proportional to their sequence lengths. The order of *LsaGRF* genes were based on the phylogenetic tree shown on the left of the figures. The size scales of gene and protein length were indicated at the bottom

To explore the phylogenic relationship of *GRF* gene family in different species, phylogenetic analysis, intron-exon and motif characteristics of *LsaGRFs* were performed. Phylogenetic analysis of *GRF* family in lettuce was firstly assessed and visualized using a Neighbor-Joining phylogenetic tree (Fig. 2A). All of the 15 *LsaGRFs* were divided into two groups (including 9 and 6 *LsaGRFs* respectively), each of which contained two small subgroups in the phylogenetic tree. Gene structure and motifs were considered to have a divergence during gene evolution. Therefore, the gene structures and motifs were listed in phylogenetic tree’s order (Fig. 2B). Most *LsaGRF* genes contain three or four exons. The *LsaGRF* genes containing the same number of exons were in the same group. For examples, *LsaGRF4* and *LsaGRF9* both have two exons. *LsaGRF11*, *1*, *2* and *13* have four exons and the rests in another group have three exons expect *LsaGRF7* (Fig. 2B). In conserved motifs analysis, motif 1 (yellow rectangle) and motif 2 (purple rectangle) were present to be the WRC and QLQ protein domain. As shown in Fig. 2B, the LsaGRF proteins in the same branch of phylogenetic tree have similar position and numbers of QLQ and WRC domains. All these analyses showed that the phylogenic relationship of *LsaGRF* genes was highly consistent with the gene structures and motif divergence of *GRF* genes in lettuce.

**Fig. 3:**
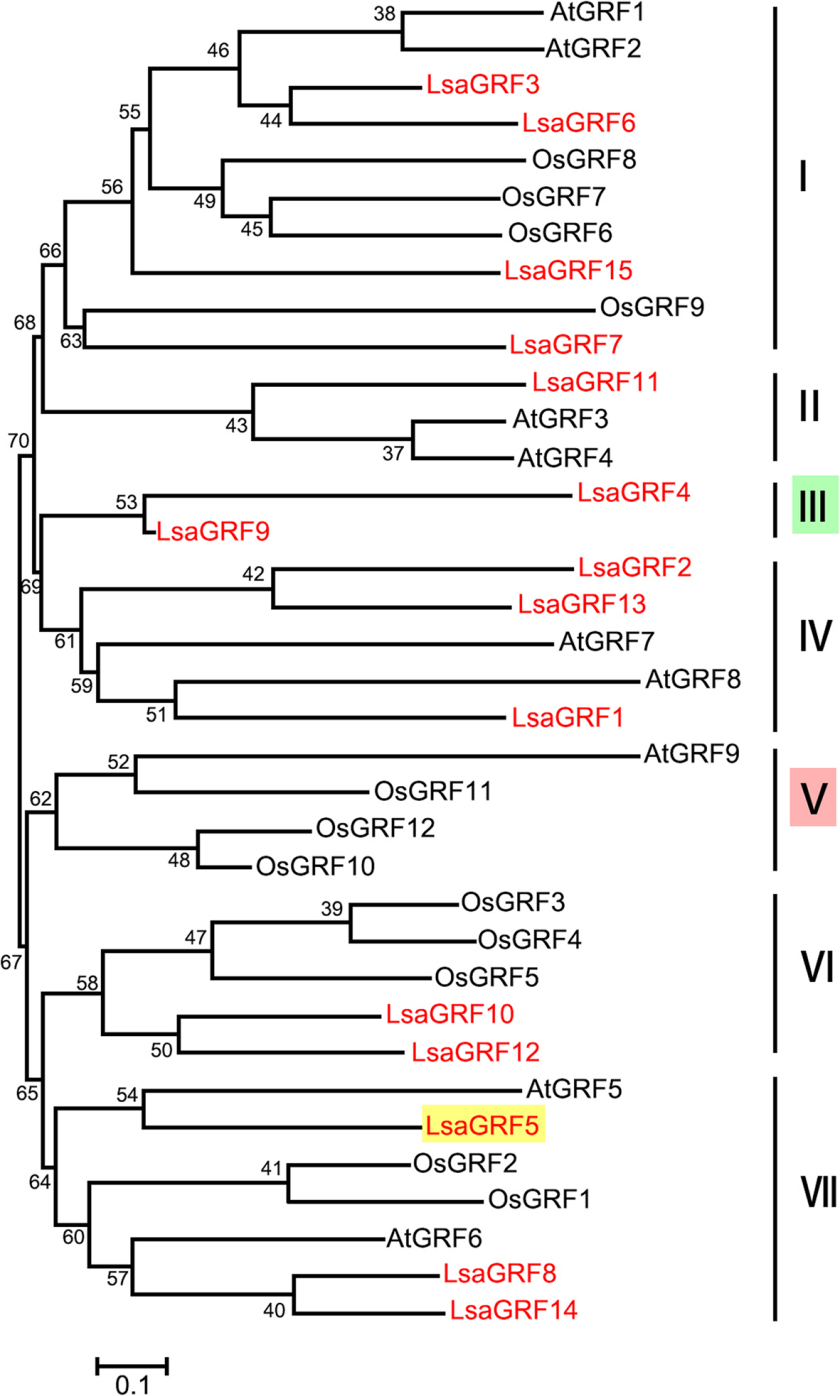
Phylogenetic relationship between GRFs from *Arabidopsis*, rice and lettuce

The functions of *GRF* genes in *Arabidopsis* and rice were extensively studied. The phylogenic relationship of *GRF* genes in lettuce, *Arabidopsis* and rice were helpful for putative function prediction of *GRF* genes in lettuce. There are totally 12 and 9 *GRF* genes in rice and *Arabidopsis*, respectively. The Neighbor-Joining phylogenetic tree of *GRF* genes from lettuce, rice and *Arabidopsis* showed that there were six groups according to the tree (Fig. 3). It was revealed that the phylogenic relationship of *GRF* genes in lettuce, rice and *Arabidopsis* were divergence. There were two groups, group I and VII, harboring *GRF* genes from lettuce, rice and *Arabidopsis*, which indicated that these *LsaGRFs* had putative orthologous genes in both rice and *Arabidopsis*. In group II and IV, there were just *LsaGRFs* and *AtGRFs*, but no *GRF* genes from rice, while there was no GRF gene from *Arabidopsis* in group VI. Group III contained only two *LsaGRFs*, *LsaGRF4* and *LsaGRF9*, but no *AtGRFs* or *OsGRFs*, while group V contained three *OsGRFs* and one *AtGRF* gene, but no *LsaGRF* gene. Interestingly, the role of *AtGRF5* could not be taken over by other members of *AtGRFs*, though there were partly overlapping functions between *AtGRFs* [3, 12]. Therefore, we chose the putative homolog gene of *AtGRF5*, *LsaGRF5*, based on the phylogenic relationship derived from phylogenic tree for further functional analysis (Fig. 3).

Phylogenetic tree was constructed for 15 *Lactuca sativa*, 9 *Arabidopsis thaliana* and 12 *Oryza sativa* GRF proteins. There were 7 phylogenetic clusters designated as I–VII. LsaGRFs were written with red fronts. The cluster III that contained GRFs only from lettuce was highlighted with green and the cluster V without LsaGRFs was shaded in red. The LsaGRF5, which was selected for further investigation, were highlighted in yellow. The scale bar represents 0.1 amino acid changes per site.

### Functional characterization of *LsaGRF5*

**Fig. 4:**
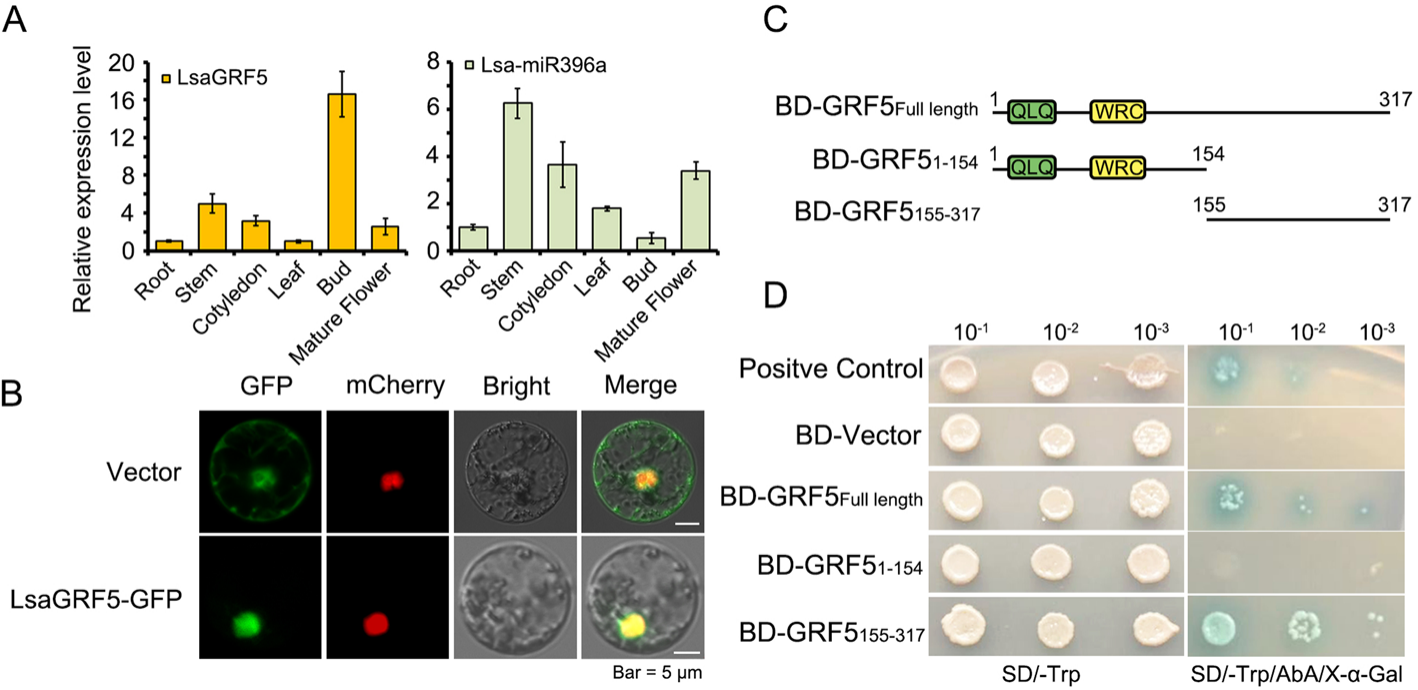
The functional characterization of *LsaGRF5* gene and characterization of LsaGRF5 protein. **A** The expression profiles of *LsaGRF5* and pri-*Lsa-MIR396a* in different tissues of the lettuce ‘YDL’. Actin was used as a reference gene. **B** Subcellular localization of LsaGRF5 in lettuce protoplasts. Confocal images showed that the fluorescence of LsaGRF5-GFP fusion protein was completely overlapped with that of Ghd7-mCherry, which was specifically expressed in the nucleus. The vector with 35S::Ghd7-mCherry only was used as a control. Bar = 5 mm. **C** The schematic diagrams of the constructions used in transactivation assay in yeast. The full-length, N-terminal region (1–154 aa) containing QLQ and WRC domains and C-terminal region (155–317 aa) of the LsasGRF5 were respectively fused into DNA sequences containing a GAL4 DNA-binding domain in pGBKT7 (BD). **D** Transactivation assay of different LsaGRF5 constructs in yeast. The constructs in (B) were expressed in the yeast strain Y2HGold with PGBDT7-OsMYB103L as a positive control and the negative control plastid (pGBKT7). The transformants with different diluted concentrates were dropped on the SD/−trp and SD/−trp/AbA/X-alpha-gal plates. After 2–4 days at 30 °C, possible transcriptional activation functions of LsaGRF5-full length and LsaGRF5_155–317_ were observed

To characterize the putative functions of *LsaGRF5*, the expression profile of *LsaGRF5* was detected. From the quantitative real-time PCR (qRT-PCR) results, the expression levels of *LsaGRF5* in roots and leaves were relatively low, while those in the bud were significantly high (Fig. 4A). However, in mature flowers with mature pollens and pistils, the expression level of *LsaGRF5* was much lower comparing that in the bud (Fig. 4A), indicating that *LsaGRF5* probably function in flower development. Meanwhile, we also detected the expressions of *Lsa-miR396a*, putatively regulating the expression of *GRF* genes, in these tissues. We found that *Lsa-miR396a* were relatively highly expressed in stem, cotyledon and mature flower, while significantly much lower in buds (Fig. 4A). The tissues with high *Lsa-miR396a* expression, e.g. mature flowers and stems, showed relatively low expression of *LsaGRF5*. And vice versa the tissues with high *LsaGRF5* expression, e.g. buds, showed relatively low expression of *Lsa-miR396a*., indicating that *LsaGRF5* might be regulated by Lsa-miR396a.

The LsaGRF proteins are putative TFs. We chose the LsaGRF5 and performed its subcellular location observation and transactivation assay. We isolated the protoplast cell of lettuce and transformed the vector containing *35S:LsaGRF5*-*GFP* and *35S:Ghd7*-*mCherry*, which was reported to locate in nuclear, into lettuce protoplast cells. The empty vector was used for control. As shown in Fig. 4B, green fluorescence of GFP and red fluorescence of mCherry were totally overlapped in the protoplast cell transformed by *35S:LsaGRF5*-*GFP*, indicating that the LsaGRF5-GFP and Ghd7-mCherry have the same nuclear localization. While the protoplast cell transformed by empty vector exhibited ubiquitous green fluorescence, excepting the overlapped region with the red nuclear fluorescence of Ghd7-mCherry (Fig. 4B). Therefore, LsaGRF5 located in the nucleus.

To identify which part of the LsaGRF5 protein had the transcriptional activity, we divided the LsaGRF5 into two parts based on the conserved protein domains. One part is the N-terminal of LsaGRF5, GRF5_1–154_, containing QLQ and WRC domains, and the other, GRF5_155–317_ (Fig. 4C). Full-length and two partial LsaGRF5s were constructed into yeast expression vector, *pGBD-T7*. The empty vector *pGBD-T7* and *pGBD-T7-OsMYB103L* which was proved to have transcriptional activity were designed as the negative and positive control, respectively [40]. These recombinant plasmids were transformed into yeast strain Y2HGold. They showed similar growth states without Tryptophan (Trp) under different diluted concentration (Fig. 4D), indicating that the recombinant plasmids were indeed transformed into the yeast cells and the transformation made few influences on the yeast growth. The yeast cells expressed full-length *GRF5* and *GRF5*_*155–317*_ could grow with AbA (Aureobasidin A) and turn blue with X-alpha-galactoside, which were the same as the positive control (Fig. 4D). These results suggested that the C-terminal contributed to the transcriptional activity of LsaGRF5, while the N-terminal containing QLQ and WRC domains did not.

### *LsaGRF5* is a miR396a target gene in lettuce

**Fig. 5:**
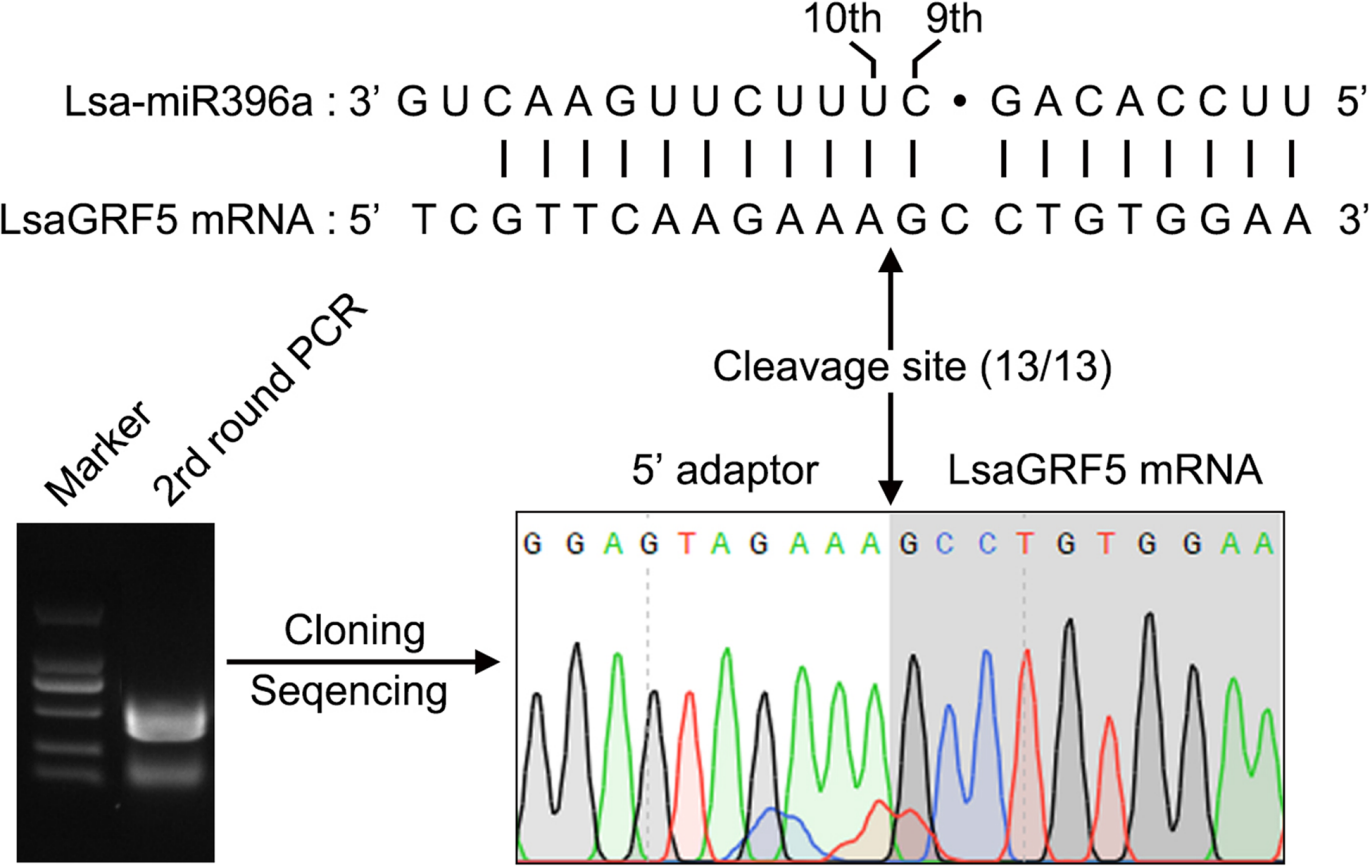
Validation of the miR396 cleavage site in *LsaGRF5* by 5′ RLM-RACE. The cleavage site of *LsaGRF5* was indicated by the arrows with the frequency of clones and sequencing peak diagram shown. The upper panel indicated the mRNA sequences alignment of miR396 and *GRF5*. 13/13 indicated that all of independent 13 assays presented the same cleavage site

The *GRF* gene family is known as the target of miR396 [29, 30]. To verify this in lettuce, we firstly predicted the complementarity between Lsa-miR396 and *LsaGRFs*. Lsa-miR396a shared nearly perfect complementarity with 14 *LsaGRFs* except *LsaGRF9* (Fig. S3). The free energies of duplex structures were all lower than − 30 kcal/mol except for *LsaGRF9* (− 30.6 kcal/mol) (Fig. S3). It means that all *LsaGRF* genes except for *LsaGRF9* probably were the targets of Lsa-miR396a. We chose *LsaGRF5* for further verification, and performed the 5′ RNA ligase-mediated (RLM) rapid amplification of cDNA ends (RACE) assay. The results showed that the 1 ~ 10 bp of the target sequence in *LsaGRF5* did not exist in the sequencing results, which means the transcript of *LsaGRF5* was cleaved at base 10 of the miR396 target site (Fig. 5). Therefore, *LsaGRF5* was probably the target of Lsa-miR396a. Function analysis in vivo could further clarify the regulatory relationship between *LsaGRF5* and Lsa-miR396.

### The phenotypes of *LsaGRF5* and Lsa-miR396 overexpression lines

**Fig. 6:**
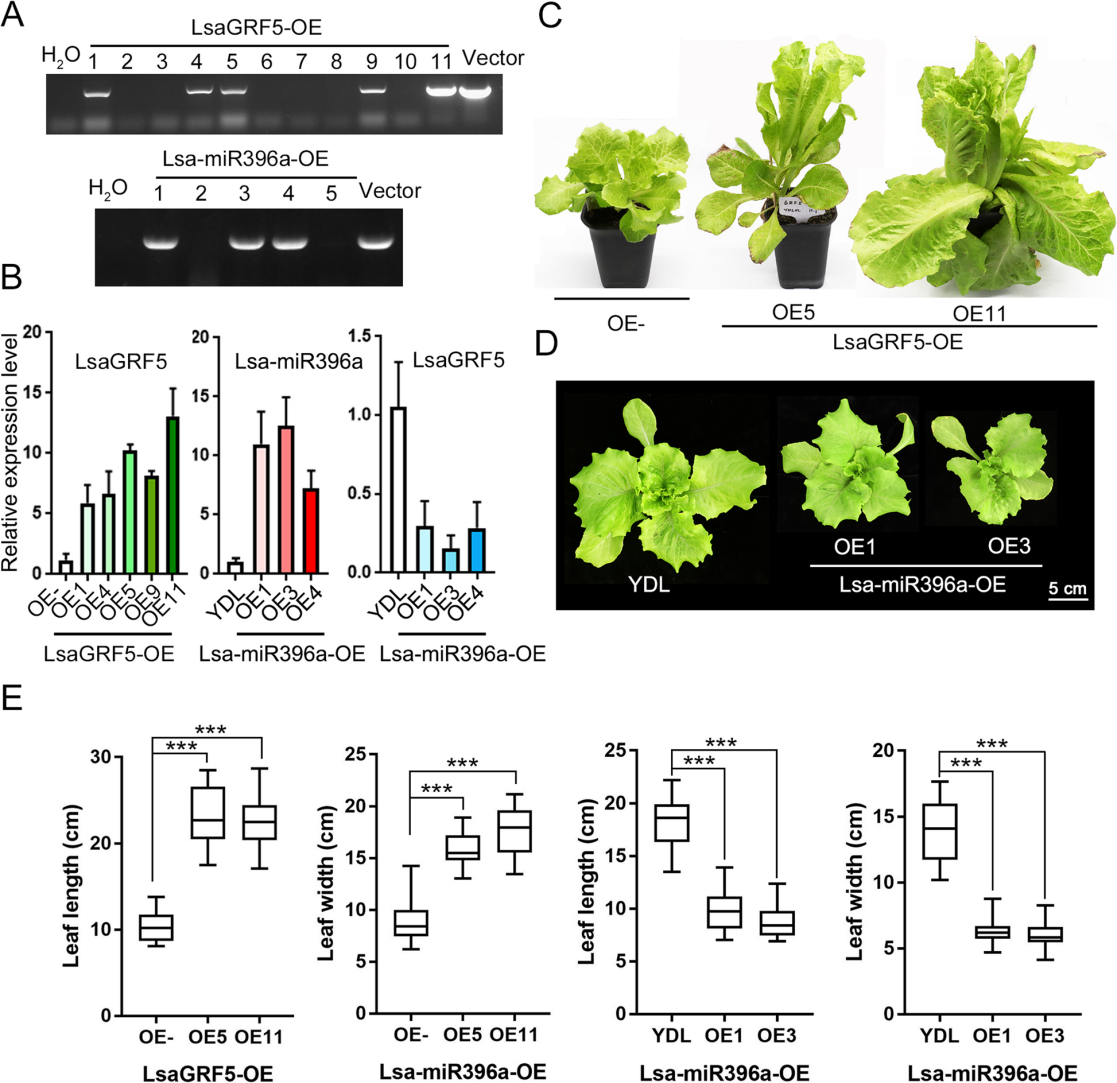
The phenotypes of overexpression lines of *LsaGRF5* and Lsa-miR396a in lettuce. **A** Verification of positive overexpression lines of LsaGRF5-OE and Lsa-miR396a-OE. The samples used double-distilled H_2_O (ddH_2_O) and transformed vector as template were designated as negative and positive control, respectively. **B** Expression assay of *LsaGRF5* and Lsa-miR396a in LsaGRF5-OE and Lsa-miR396a-OE lines. The expression level of one of three biological replications in negative control was defined as “1”. Values represent means ± SD (*n* = 3) from three biological replicates. **C** The phenotype of T_1_ progeny of LsaGRF5-OE. The seeds of T1 progeny of *LsaGRF5* overexpression line and empty vector transgenic line (OE-) were screened on the plates containing Kanamycin. OE- was used as a negative control. **D** The phenotype of T_1_ progeny of Lsa-miR396a-OE. The seeds of T1 progeny of Lsa-miR396a overexpression line and wild type lettuce ‘YDL’ were sowed and planted in greenhouse at the same time. ‘YDL’ was used as a negative control. e. Leaf length and width of *LsaGRF5*, *Lsa-miR396a* overexpression lines and their controls at the 10-leaf stage. The outermost leaves were subjected to measurements. Box plots display median (line), interquartile range (box), and whiskers (extending 1.5 times the interquartile range). *n* > 30. The statistical significances were determined using Student’s *t* test. ***P < 0.001

To investigate the function of *LsaGRF5*, we constructed the overexpression lines in lettuce. *LsaGRF5* driven by CaMV 35S promoter was transformed into the lettuce cultivar of Romaine type ‘YIDALI’ (YDL). Eleven independent transgenic lines were obtained. Transformation verification was carried out through a pair of primers located on 35S promoter and *LsaGRF5*, respectively. The results showed that five out of eleven were positive transgenic lines with the same band as the positive control (Fig. 6A). qRT-PCR assay revealed that the expression of *LsaGRF5* increased 5 ~ 15 folds in these five lines (Fig. 6B). Two lines, LsaGRF5-OE5 and LsaGRF5-OE11, with high *LsaGRF5* expressions were used for further phenotypic analysis. The leaves of LsaGRF5-OE5 and LsaGRF5-OE11 were significantly bigger than these of YDL transformed by empty vector (OE-) (Fig. 6C). The bigger leaves also existed in *LsaGRF5* overexpressed in *Arabidopsis* (Fig. S4). From these results, *LsaGRF5* could enhance the leaf growth, and this function was conserved in *Arabidopsis.*

To figure out the functional relevance between Lsa-miR396a and *LsaGRF5*, we also overexpressed *Lsa-MIR396a* in YDL. Three positive transgenic lines were obtained by PCR of genomic DNA (Fig. 6A). The transcriptional levels of Lsa-miR396a were about 8 ~ 15 times higher in overexpression lines compared with negative control (Fig. 6B). Two overexpression lines with high expression level of Lsa-miR396a showed smaller leaves, the opposite phenotype of LsaGRF5-OE (Fig. 6C). We detected the expression level of *LsaGRF5* in Lsa-miR396-OE lines, and the results revealed that they were suppressed in Lsa-miR396a-OE lines (Fig. 6D). To quantify the leaf growth changes in these transgenic lines, we measured the length and width of the outermost leaves at 10-leaf stage. The leaves in *LsaGRF5* overexpression lines were significantly larger than these of YDL transformed by empty vector (OE-) both in length and width (Fig. 6E and Table S2). However, the leaf length and width of *Lsa-miR396a* overexpression lines were significantly smaller than these of wide type ‘YDL’ (Fig. 6E and Table S2). These results suggested that *LsaGRF5* was identified as a regulatory factor in leaf size, while Lsa-miR396a was also found to function in leaf size by the regulation of expression of *LsaGRF5*.

## Discussion

GROWTH-REGULATING FACTOR, a type of plant specific transcription factor, plays important roles in plant growth and development. Our studies focus on the genome organization, conservation and function of GRFs in lettuce. 15 *GRF* genes were identified based on the recently released genome data of lettuce cultivar ‘Salinas’ [39]. The regulatory genes are considered to be preferentially retained after genome duplications [41]. The GRF gene family has subjected to two expansions with one occurred through the whole-genome triplication in the common ancestor of eudicots and the other one occurred during independent whole-genome duplications in various plants [8]. Among the 15 identified *LsaGRFs*, not all *LsaGRF* genes contained both QLQ and WRC domains. LsaGRF7 contains only WRC domain, and LsaGRF4 contains two WRC domains but no QLQ domain, which might be the results from whole-genome duplication and recombination, and might play distinct roles in plant growth regulation (Fig. 2B). Interestingly, LsaGRF9, like rice and maize GRF10, had truncated C-terminal [14, 16, 42]. Overexpression of *ZmGRF10* may break the homeostasis of GRF/GIF (GRF INTERACTION FACTOR) to affect leaf growth, whether or not *LsaGRF9* functioned in the same way needs to be further addressed [42].

Through subcellular location and transactivation assay, LsaGRF5 probably worked as transcriptional factor in cell nucleus. Notably, AtGRF5 was proved to be able to interact with GIF1 to regulate cell proliferation in leaf primordium [3]. In addition, GIF proteins have recently been reported to play a role in transcription regulation not only by the interactions with GRFs but also with various chromatin remodeling proteins [12, 43]. Whether there were functional *GIF* genes in lettuce, and if LsaGRF5 could interact with GIF to regulate leaf growth needs further investigation. It is already known that miR396 directly cleaves the GRF genes on their complementary sequence to suppress their expression [30]. In this study, we firstly predicted that there were 14 *GRF* genes, except for *LsaGRF9*, as the putative targets of Lsa-miR396 based on the free energies of duplex structures analysis. The cleavage sites of *LsaGRF5* were confirmed by 5′ RACE in vivo. At the same time, the expression of *LsaGRF5* was significantly decreased in Lsa-miR396a-OE lines. Among nine *GRF* genes in *Arabidopsis*, *AtGRF5* and *AtGRF6* were found not to be the target of miR396 [29, 30]. *AtGRF5*, *AtGRF6* and *LsaGRF5* belonged to group VII, while *LsaGRF9* was in group III (Fig. 3), suggesting that the regulation pattern of miR396-GRFs might be distinct in lettuce and *Arabidopsis thaliana*. Recently, *AtGRF5* was found to function in chloroplast development, nitrogen signaling and senescence, besides leaf development [44]. Therefore, besides some conserved function characteristic of *GRF* genes, it is valuable to know whether *LsaGRF5* has other functions.

Expression profile of genes would help us to predict their potential biological function. The expression patterns of *GRFs* have been previously investigated. They usually express in growing zones of roots and shoots where cell proliferation occurs [2, 3, 10, 11, 13, 15]. Here, we detected the expression of *LsaGRF5* in root, stem, cotyledon, leaf, bud and mature flower, and revealed that *LsaGRF5* was highly expressed in bud where cell proliferation occurs violently. Moreover, expression level of *AtGRF* is suppressed during plant aging [2, 30]. The expression of *LsaGRF5* in bud was significantly higher than that in mature flower, which is consistent with previous results that *GRFs* functioned in the early stages of the growth and development in different tissues [2, 3, 30]. In previous researches, the strong expressions of all *AtGRF* genes were found in the shoot apical region and flower buds, where no morphological changes were observed [2]. Instead, the alterations in leaf growth and development were detected, though the expression levels of *AtGRF* genes were very low in leaves [2]. These results were consistent with these in this study. All *AtGRF* genes have low expression level in leaf and high in root, bud and mature flower, which is consistent with our expression results of LsaGRF5 in our manuscript [2]. The expression patterns of other *LsaGRF* genes should be detected in future work. The overexpressed *AtGRF5* could increase the leaf area by increasing the cell number, but not cell area [3]. The *LsaGRF5* overexpression lines also exhibited the larger leaves, which caused by increased cell number or area should be deeply investigated.

## Conclusion

In this study, we firstly identified all of the *GRF* gene family members in lettuce. The phylogenic relationship of these GRFs genes in lettuce with their counterparts in *Arabidopsis thaliana* and rice and conserved motif were evaluated. *GRFs* were well-known as target genes of miR396s. Therefore, thus is a daily use, we also characterized the chromosomal location of the stem-loop sequences features of Lsa-miR396s. Furthermore, *LsaGRF5* could probably function as a transcriptional factor in cell nucleus through subcellular location observation and transactivation assay. Overexpression of *LsaGRF5* could stimulate the leaf growth leading to bigger leaves, while overexpression of Lsa-miR396a exhibited smaller leaves with suppressed expression of *LsaGRF5*. In summary, the expression of *LsaGRF5* was regulated by Lsa-miR396 through the cleavage of complementary sequences to control leaf growth. Our findings will facilitate further understanding of the functions of *GRF* genes and help elucidating the leaf development mechanism in lettuce.

## Methods

### Plant materials and growth conditions

The lettuce (*Lactuca sativa* L.) cultivar of Romaine type, cv. ‘YIDALI’ (YDL), was used for transformation in this study. ‘YIDALI’ (YDL) was a commercial variety cultivated by Beijing Vegetable Research Center and the seeds were also provided by Beijing Vegetable Research Center, Beijing Academy of Agriculture and Forestry Science, Beijing, China. The sterilized lettuce seeds were grown on Murashige and Skoog (MS) medium plus 3% sucrose and 0.6% agar (pH 5.8) at 25 °C in a 16-h-light /8-h-dark cycle. The full expanded cotyledons were used for transformation. The transgenic lettuce plants were grown in a growth chamber under a photoperiod of 16-h light (200 μmol m^− 2^ s^− 1^) and 8-h dark at 25 °C. When the fifth true leaf was fully expanded, the lettuce plants were transplanted into a greenhouse in Beijing Vegetable Research Center under standard greenhouse conditions.

For lettuce transgenic lines, we collected the seeds of T1 progeny from T0 seedlings, which were grown in a growth chamber described above. The T1 seeds of GRF5 overexpression lines were screened on the plate containing Kanamycin. The T1 generation of transgenic lines transformed with the empty vector (OE-) were used as the control and the seeds of it were screened simultaneously. And then the seedlings were transferred to the same size pots for genotyping. The T1 seeds of Lsa-miR396a overexpression lines and wide-type control ‘YDL’ were sown directly in soil in the greenhouse described above without antibiotic screening and then we detected the transformation positive lines using genomic PCR and qRT-PCR. The phenotypes of transgenic positive and wide-type lines were observed in greenhouse.

### Identification of *GRF* genes in lettuce

The genome sequences of lettuce (*Lactuca sativa* V8) were downloaded from the Phytozome (https://phytozome.jgi.doe.gov/pz/portal.html). The sequences of Lsa-miR396s were obtained from PmiREN (Plant miRNA Encyclopedia, http://www.pmiren.com) [45]. The sequences of *AtGRFs* and *OsGRFs* were retrieved from the *Arabidopsis thaliana* Information Resource (http://www.arabidopsis.org/) and China Rice Data Centre (http://www.ricedata.cn/) respectively. The amino acid and nucleotide sequences of AtGRFs and OsGRFs were used for BLASTP and BLASTN (E < 0.01) searching in Phytozome (*Lactuca sativa* V8) to obtain a list of putative *LsaGRF* genes. Subsequently, the amino acid sequences of the obtained putative *LsaGRFs* were reiteratively used for BLAST searching. The conserved protein domains in the GRF proteins, including QLQ (PF08880) and WRC (PF08879), were used for searching in the lettuce Protein Database in GRAMENE [46], and the protein domains of LsaGRFs were confirmed in the Pfam database (http://pfam.sanger.ac.uk/)(E-value < 1 × 10^− 4^) [47]. Finally, the results were supplemented using the HMMER software.

### Characterization of LsaGRFs and miR396s

The molecular masses of the putative GRF proteins were calculated using the Compute pI/Mw tool of ExPaSy (http://web.expasy.org/compute_pi/). Schematic *LsaGRF* gene structure diagrams were drawn using the Gene Structure Display Server (http://gsds.cbi.pku.edu.cn/). Protein sequence motifs were predicted using the MEME program (http://meme.sdsc.edu/meme/). The physical position of each LsaGRF gene on the ten Chinese cabbage chromosomes was determined from the Chinese cabbage database (BRAD, http://Chinesecabbagedb.org/brad/) and marked on each chromosome using the MapInspect software (http://mapinspect.software.informer.com). The combination of phylogenetic tree, gene, and protein structures was generated using the iTOL tool (http://itol.embl.de) [48].

### Phylogenetic analysis

To construct the phylogeny of the GRFs from various species, multiple sequence alignments for all GRF amino acid sequences were conducted using MEGA 7.0 with default settings [49]. Phylogenetic analyses were carried out with a Neighbor-Joining method using MEGA 7.0 [49].

### Lettuce protoplast isolation and subcellular location of LsaGRF5

The coding sequence of *LsaGRF5* was cloned into the pSAT6-EGFP-N1 vector. The 35S:LsaGRF5-GFP and 35S:GFP plasmids (1 μg/μL, 5 μL each) were transformed into protoplasts of the lettuce ‘YDL’ by means of polyethylene glycol treatment [50]. Transformed protoplasts were observed using a fluorescence microscope (Leica TCS SP5). Images were analyzed with Image LAS-AF software. *Ghd7*-mCherry was used as controls for nuclear [51]. Primers used were listed in Table S3.

### Transactivation assay based on the yeast GAL4 system

The transcriptional activity of LsaGRF5 was evaluated in yeast cells. The full-length coding sequence, N-terminal LsaGRF5 DNA-binding domain (1–154 aa) and the C-terminal putative activation domain (155–317 aa) were (respectively) cloned into pBD-GAL4vector. The empty vectors pGBKT7 and GAL4 were used as negative and positive controls, respectively. All of these constructs were individually introduced into cells of yeast strain Y2HGold containing the AUR1-C and MEL1 reporter genes. Yeast cell transformation was carried out using the instructions in the Yeast maker™ Yeast Transformation System 2 User Manual. The yeast transformants were grown on SD/−Trp and SD/−Trp/ in the presence of Aba and X-alpha-gal plates for 2–4 d at 30 °C to identify transactivation activity (Yeast Protocols Handbook; Clontech, Mountain View, CA, USA). Primers used were showed in Table S3.

### RNA extraction and qRT-PCR

Total RNA was extracted from the leaves using a plant RNeasy kit (Tiangen, Beijing, China). RNA was reverse transcribed into cDNA with a PrimeScript™ RT reagent Kit (Takara, Osaka, Japan). Real-time PCR reactions were performed using the SYBR Green I Master Mix and were quantified in a Light Cycler 480 II instrument (Roche, Basel, Switzerland). The PCR program comprised an initial step at 94 °C for 30 s, followed by 40 cycles of 94 °C for 10 s and 58 °C for 30 s. Amplification was followed by heating for 1 min at 60–95 °C for melting curve analysis. Each reaction was performed with three replications using 5 μL of Master Mix, 0.25 μM of each primer, 1 μL of diluted cDNA, and DNase-free water to a final volume of 10 μL. Three biological replicates were collected for each sample. According to Yu et al. 2020 [52], the lettuce actin genes were used as internal controls to normalize the transcript levels of target genes. Relative gene quantification was calculated by the comparative ΔΔCt method [53]. The average 2 − ΔΔCt values were used to determine differences in gene transcript levels. The lengths of PCR products were among 300 to 500 bp. The PCR products were sequenced in a commercial DNA sequencing company. The primers were designed using the Primer Premier 6.0 software and were shown in Table S3.

### Plasmid construction and plant transformation

The cloning primers of *LsaGRF5* and *Lsa-MIR396a* were designed based on the genome sequences of lettuce (*Lactuca sativa* V8) from the Phytozome (https://phytozome.jgi.doe.gov/pz/portal.html). The coding sequences of *LsaGRF5* and *Lsa-MIR396a* were cloned from the lettuce ‘YDL’ and constructed into the overexpression vector pEZR(K)-LC [54] driven by 35S promoter using 2 X Seamless Cloning Mix (Biomed, CL117–01, Beijing). The primers used for vector construction were listed in Table S3.

The recombinant constructs were transformed into Lettuce using Agrobacterium-mediated transformation following the leaf disk method [55]. The seeds sterilized with 10% bleach were sowed on Murashige and Skoog MS media at 22 °C, 16-h-light/8-h-dark, in a growth chamber. After 5 days, cotyledons were cut and immersed in a suspension of Agrobacterium (OD_600_ = 0.6) for 15 min and then were transferred to the co-cultivation media (MS with 1 mM acetosyringone) in the dark for 2 days. The co-cultivated cotyledons were subjected to the selection and shoot-inducing media (1x MS, 0.1 mg/L 1-naphthlcetic acid, 0.1 mg/L 6-BA, 50 mg/L Kan, 300 mg/L Timentin) at 22 °C for about 2 weeks. The young buds were transferred to the root-inducing media (MS, 300 mg/L Timentin). Finally, the resistant seedlings were transferred to soil and further verified by PCR and qRT-PCR.

### MiR396 cleavage site analysis

A 5′ RLM-RACE was used to detect the miR396 cleavage site in miR396 target genes and was performed accordingly [56]. Total RNA of ‘YDL’ leaves was extracted using a plant RNeasy kit (Tiangen, Beijing, China). Then 5′ adaptor ligation RNA was prepared according to the kit (NEB, M0204, MA, USA). The fist chain cDNA was synthesized based on PrimeScript™ RT reagent Kit kit (Takara, RR047, Osaka, Japan). Furtherly, the RLM-RACE reactions were amplified with the enzyme TKS (Takara, Cat# AI51320A, Osaka, Japan) and 2 X TransStart FastPfu PCR SuperMix (TransGene, Cat# AS221–01, Beijing, China), respectively. Finally, the target DNA fragment was purified and cloned into vector using pEASY-Blunt Zero Cloning Kit (TransGene, CB501–01, Beijing, China). At least 15 positive clones of each gene were picked for sequencing. The gene-specific primers (GSPs) and 5’adaptor sequences were listed in Table S3.

## Supplementary Information

**Additional file 1: Figure S1.** The amino acid sequences alignment of LsaGRF genes. **Figure S2.** Phylogenetic analysis of Lsa-miR396s. **Figure S3.** The degree of Lsa-miR396a complementarity to all LsaGRFs. **Figure S4.** The phenotypes of overexpression lines of LsaGRF5 in Arabidopsis

**Additional file 2: Table S1.** Number of GRF genes and miR396s have been identified in different species

**Additional file 3: Table S2.** The length and width of *LsaGRF5*-*OE* and *Lsa-miR396a*-*OE* leaves

**Additional file 4: Table S3.** The sequences of Primers used in this study

**Additional file 5.** The original gel images used in this article

## Data Availability

The genome and protein sequences of lettuce (*Lactuca sativa* V8) were downloaded from the Phytozome (https://phytozome.jgi.doe.gov/pz/portal.html). The sequences of Lsa-miR396s were obtained from PmiREN (Plant miRNA Encyclopedia, http://www.pmiren.com). All of the datasets supporting the results of this article are included within the article and its additional files. The sequences of *AtGRFs* and *OsGRFs* were retrieved from the *Arabidopsis thaliana* Information Resource (http://www.arabidopsis.org/) and China Rice Data Centre (http://www.ricedata.cn/) respectively. The sequences, alignments and phylogeny data were uploaded to the TreeBASE repository (https://www.treebase.org/). The accession number is 28704 and the data could be checked on the website: http://purl.org/phylo/treebase/phylows/study/TB2:S28704.

## Abbreviations

MW: Molecular weight
GRFs: GROWTH-REGULATING FACTORs
GA: Gibberellic acid
TF: Transcription factor
SAM: Shoot apical meristems
Ad-Ab: Adaxial–abaxial
GIFs: GRF-Interacting Factors
AbA: Aureobasidin A
MS: Murashige and Skoog
pI: Isoelectric point
qRT–PCR: Quantitative real-time polymerase chain reaction
5′ RLM RACE assay: 5′ RNA ligase-mediated rapid amplification of cDNA ends assay
