# Supplementary material for: Identification of GROWTH-REGULATING FACTOR transcription factors in lettuce (Lactuca sativa) genome and functional analysis of LsaGRF5 in leaf size regulation
Source: BMC Plant Biol. 2021 Oct 23;21:485. doi: 10.1186/s12870-021-03261-6 (PMC8539887; doi:10.1186/s12870-021-03261-6)
Supplement: Supplementary file 1 — Additional file 1: Figure S1. The amino acid sequences alignment of LsaGRF genes. Figure S2. Phylogenetic analysis of Lsa-miR396s. Figure S3. The degree of Lsa-miR396a complementarity to all LsaGRFs. Figure S4. The phenotypes of overexpression lines of LsaGRF5 in Arabidopsis [file 12870_2021_3261_MOESM1_ESM.docx]

Supplementary Material

**Identification of GROWTH-REGULATING FACTOR transcription factors in lettuce (*Lactuca sativa*) genome and functional analysis of *LsaGRF5* in leaf size regulation**

Bin Zhang^†^, Yanan Tong^†^, Kangsheng Luo, Zhaodong Zhai, Xue Liu, Zhenying Shi, Dechun Zhang*, Dayong Li*

*** Correspondence:** Dayong Li ([lidayong@nercv.org](mailto:lidayong@nercv.org) ); Dechun Zhang ([zhangdc227@163.com](mailto:zhangdc227@163.com)).

# Supplementary Figures and Tables

## Supplementary Figures


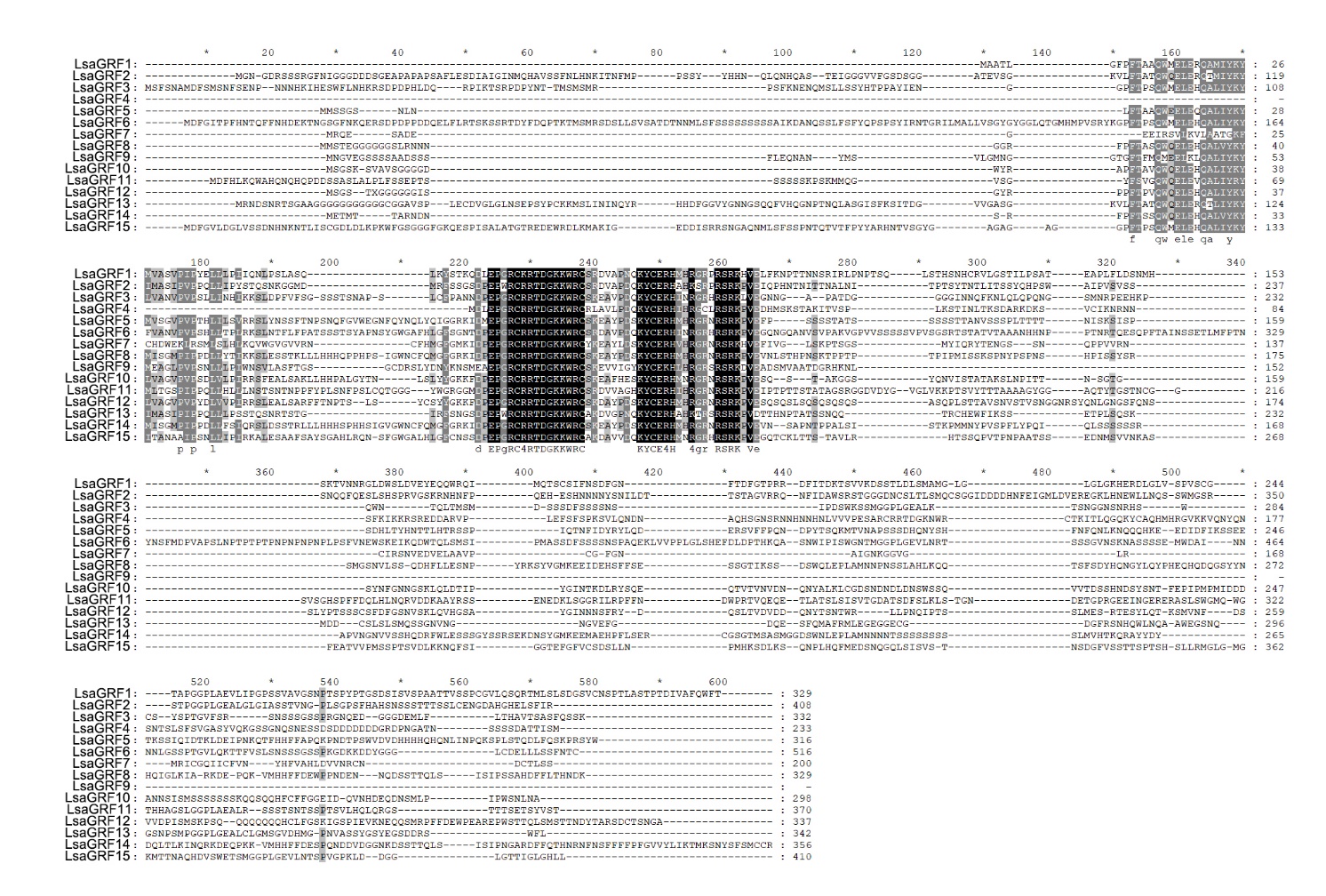


**Supplementary Figure 1. The amino acid sequences alignment of LsaGRF genes.**


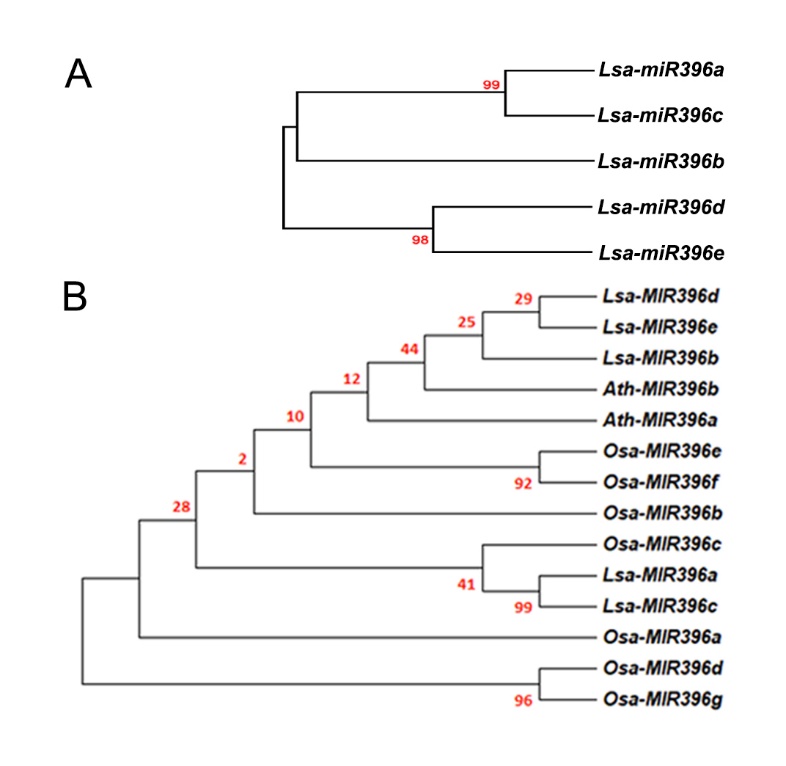


**Supplementary Figure 2.** **Phylogenetic analysis of Lsa-miR396s.**

A. Phylogenetic analysis of miR396s in lettuce. B. Phylogenetic relationship of miR396s in Arabidopsis, rice and lettuce.


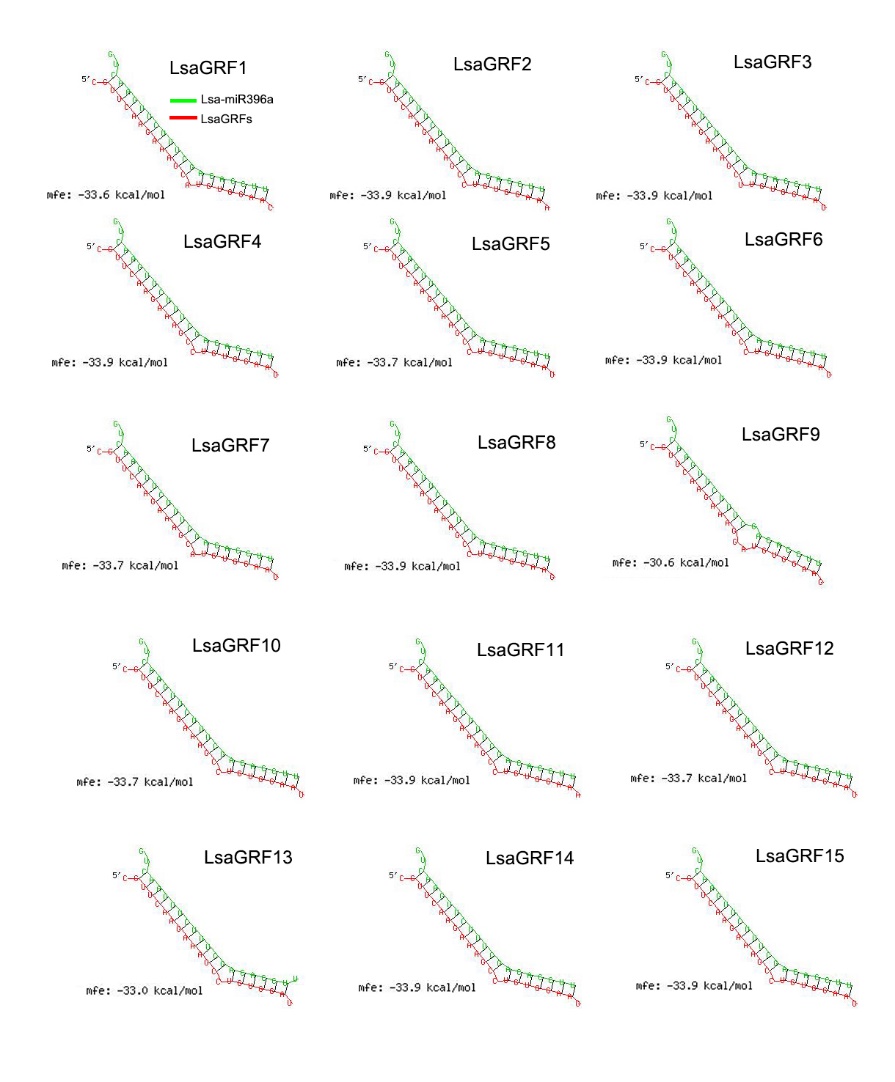


**Supplementary Figure 3. The degree of Lsa-miR396a complementarity to all LsaGRFs.** Free energies of duplex structures were calculated using RNAhybrid software (http://bibiserv.techfak.uni-bielefeld.de/rnahybrid).


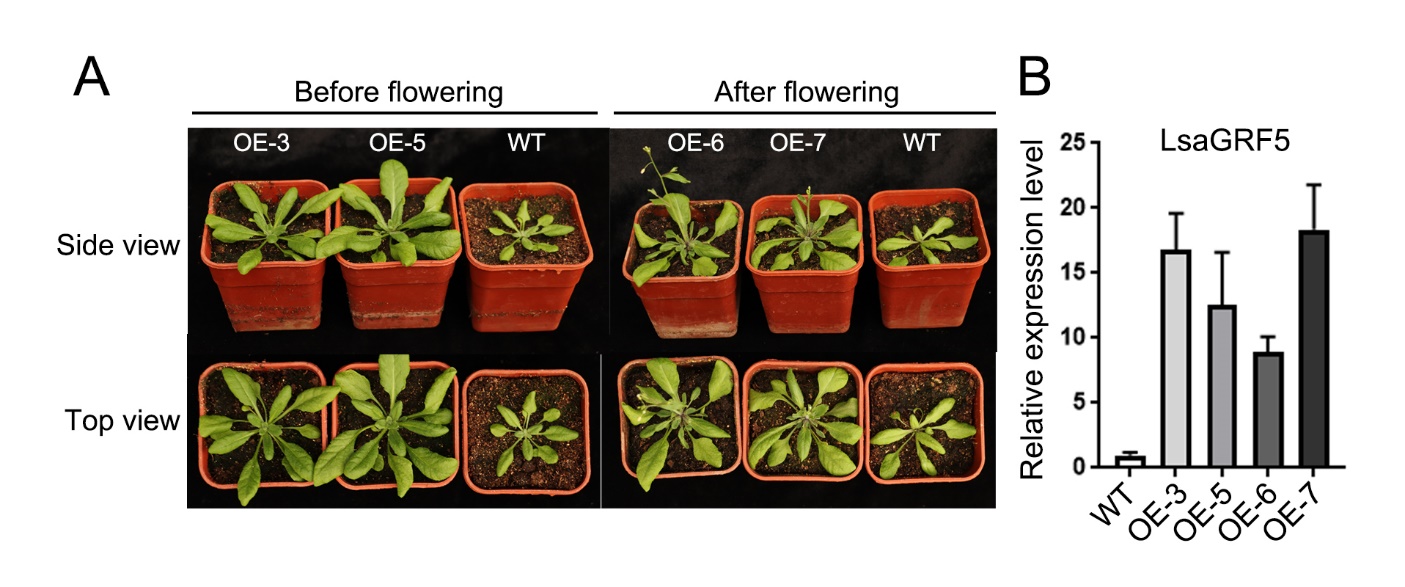


**Supplementary Figure 4. The phenotypes of** **overexpression lines of LsaGRF5 in Arabidopsis.** A. The Arabidopsis overexpression lines of LsaGRF5 showed bigger leaves and early flowering phenotypes. B. The expression level of LsaGRF5 in Arabidopsis overexpression lines of LsaGRF5.

1.1 Supplementary Tables

The Supplementary Tables for this article can be found in the attached file with excel format.
