# Supplementary material for: Identification of GROWTH-REGULATING FACTOR transcription factors in lettuce (Lactuca sativa) genome and functional analysis of LsaGRF5 in leaf size regulation
Source: BMC Plant Biol. 2021 Oct 23;21:485. doi: 10.1186/s12870-021-03261-6 (PMC8539887; doi:10.1186/s12870-021-03261-6)
Supplement: Supplementary file 5 — Additional file 5. The original gel images used in this article [file 12870_2021_3261_MOESM5_ESM.docx]

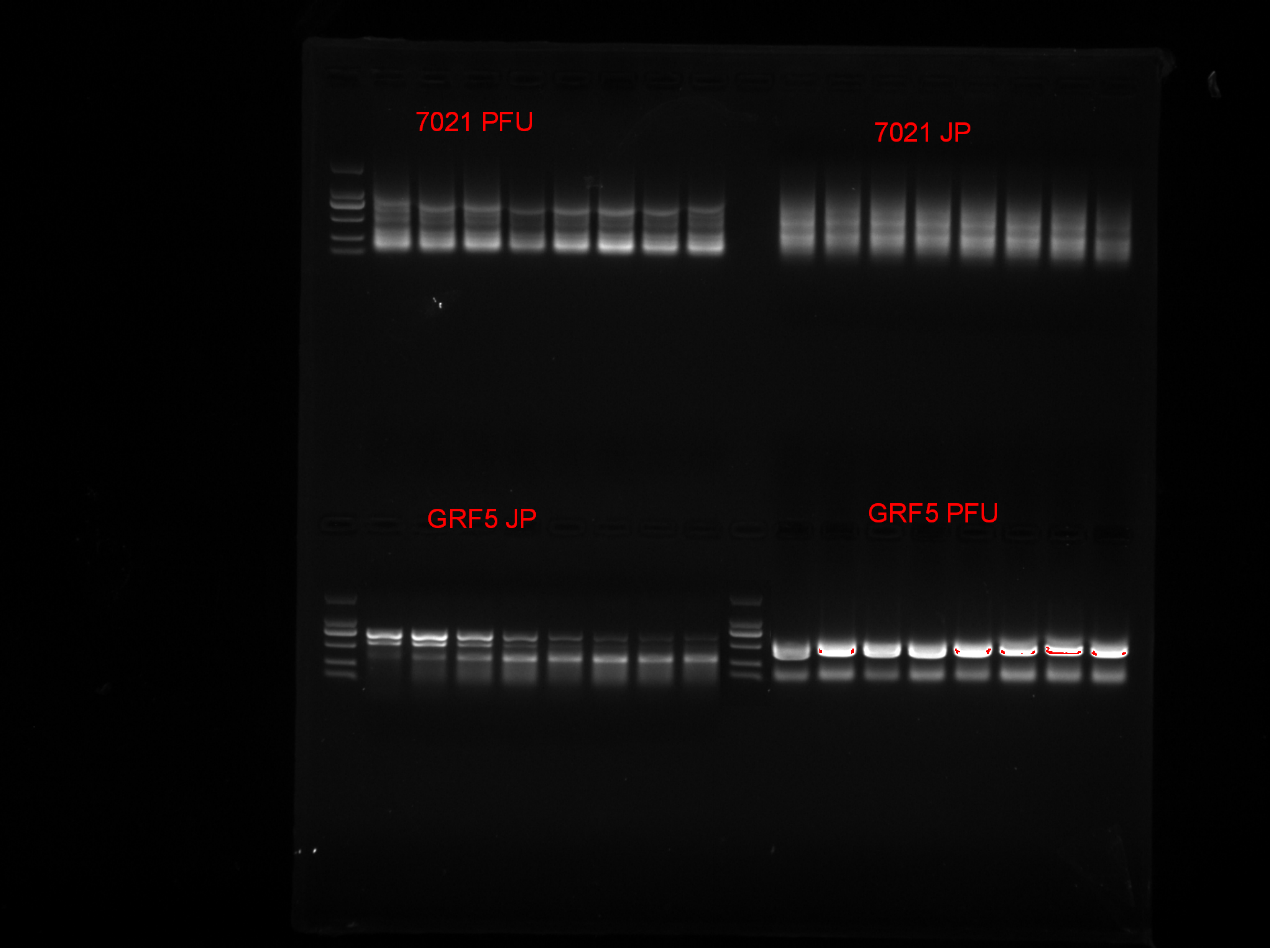


Image 1. The original gel picture of Figure 5 in the manuscript.

The image containing DNA marker and 5’ RLM RACE bands of *LsaGRF5* in the red rectangle, which could be edited and removed in word, was used in Figure 5.


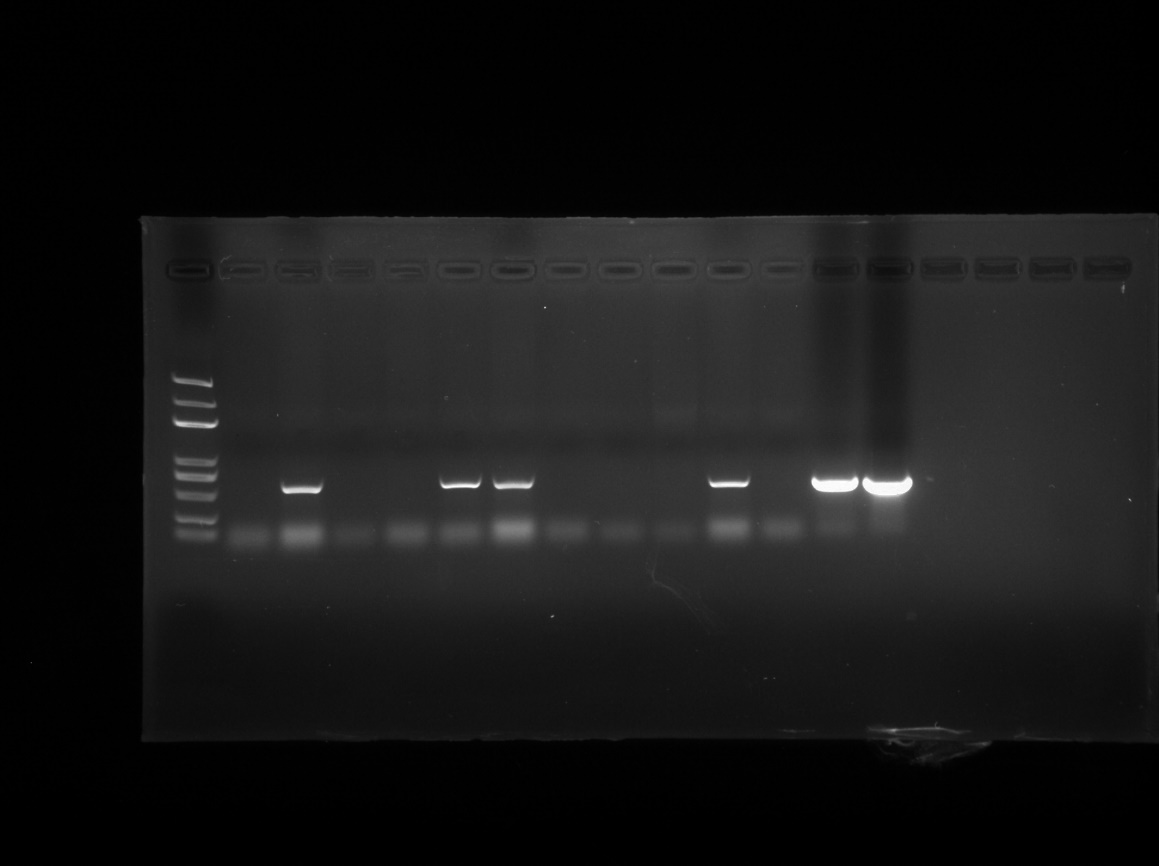


LsaGRF5-OE

H_2_O 1 2 3 4 5 6 7 8 9 10 11 Vector

Image 2. The original gel picture of Figure 6A upper panel in the manuscript.

Verification of positive overexpression lines of LsaGRF5-OE by PCR. The samples used ddH_2_O and transformed vector as template were designated as negative and positive control, respectivel


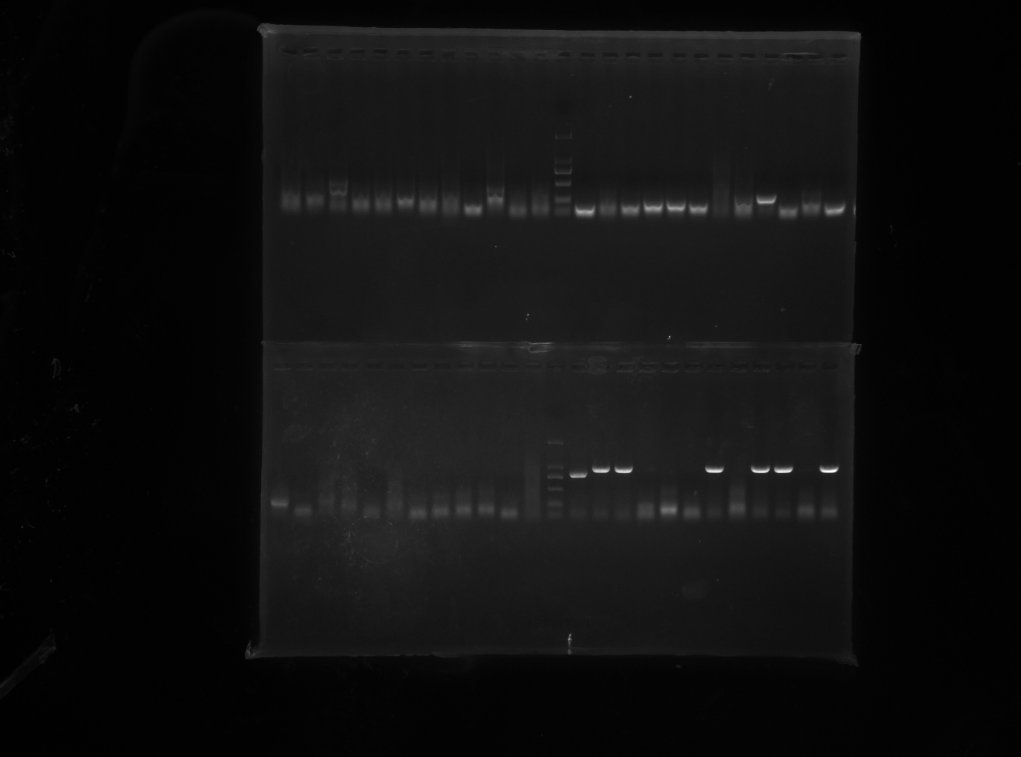


Lsa-miR396a-OE

H_2_O 1 2 3 4 5 Vector

Image 3. The original gel picture of Figure 6A lower panel in the manuscript.

The last seven lanes in the red rectangle, which could be edited and removed in word, were used in Figure 6A lower panel. Verification of positive overexpression lines of Lsa-miR396a-OE by PCR. The samples used ddH_2_O and transformed vector as template were designated as negative and positive control, respectivel
